# Identification of risk factors for acute kidney injury after pulmonary endarterectomy with cardiopulmonary bypass

**DOI:** 10.1186/s13019-020-01152-9

**Published:** 2020-05-15

**Authors:** Peng Dong, Fu-Shan Xue, Shao-Hua Liu

**Affiliations:** Department of Anesthesiology, Beijing Friendship Hospital, Capital Medical University, No. 95 Yong-An Road, Xi-Cheng District, Beijing, 100050 People’s Republic of China

**Keywords:** Acute kidney injury, Cardiopulmonary bypass, Risk factors, Postoperative outcomes

## Abstract

The letter to the editor made several comments on possible issues in the recent article by Zhang et al. determining the risk factors of acute kidney injury after pulmonary endarterectomy with cardiopulmonary bypass, which has been published in *Journal of Cardiothoracic Surgery* at December 30, 2019.

**To the Editor:**

We read with great interest the recent article by Zhang et al. [1] determining the risk factors of acute kidney injury (AKI) after pulmonary endarterectomy with cardiopulmonary bypass (CPB). By univariate and multivariate logistic regression analyses, they showed that preoperative platelet count and hemoglobin level, and deep hypothermic circulatory arrest time were the independent predictors of postoperative AKI. Other than the limitations described by the authors in discussion, however, we noted several issues in this study that were needed further clarifications.

First, 12 (9.8%) patients were defined as preoperative renal dysfunction only based on the serum creatinine (SCr) level of greater than 1.2 mg/dL. It must be emphasized that alone use of this variable to assess baseline renal function has a limited value, as many factors can significantly affect preoperative SCr levels. The available evidence indicates that preoperative occult renal dysfunction, a most established risk factor for postoperative AKI, is common among patients with a normal SCr level, with a prevalence of 49.1% [2]. In clinical practice, the useful and reliable methods to determine preoperative occult renal dysfunction actually are combined use of SCr levels and SCr clearance, and use of estimated glomerular filtration rate criteria of the National Kidney Foundation Kidney Disease Outcomes Quality Initiative Classification. We were concerned that alone use of the SCr levels to assess preoperative renal function would have underestimated the incidence of occult renal dysfunction in this study, resulting in the possibility that an important risk factor for postoperative AKI was not demonstrated to be significant in this study.

Second, the readers were not provided with intraoperative and postoperative fluid volumes and fluid balance, though perioperative fluid overload is frequent among patients with CPB surgery and has been independently associated with an increased risk of postoperative AKI [3]. Most important, moreover, it was unclear whether the SCr levels used for difination of postoperative AKI had been adjusted based on periostoperative fluid balance. As a positive fluid balance may dilute SCr levels, not adjusting SCr levels for periostoperative fluid balance can underestimate incidence and severity of AKI after CPB surgery. The recent evidence indicates that a small increase of early postoperative SCr levels adjusted for fluid balance after CPB surgery can significantly improve diagnosis and severity classifications of AKI defined by the Kidney Disease: Improving Global Outcomes criteria used in this study [4].

Third, the authors did not provide the details of intraoperative hemodynamic instability, hemoglobin levels, perfusion pressure and rate during CPB, and intraoperative use of vasopressin. In fact, both hypoperfusion and low mean arterial pressure during CPB are the most established risk factors for postoperative AKI in patients undergoing cardiac surgery [5]. Furthermore, both hemodilution anemia and hypotension during CPB can synergistically act to increase the risk of postoperative AKI [6]. In addition, an increased dose of vasopressin during surgery has been associated strongly with an increased risk of AKI after cardiac surgery [3].

Fourth, a 7-day observed period was used for determination of AKI and early postoperative adverse events were well noted in this study. Other than postoperative nadir hemoglobin level, however, most of early postoperative adverse events associated with the occurrence of postoperative AKI seemed not to be taken into the univariate and multivariate models for statistical adjustment. It is generally believed that low cardiac output syndrome, hemorrhage requiring reexploration, sepsis, infection, pulmonary complications, hypotension and blood transfusion in early postoperative period are significantly associated with an increased risk of AKI after CPB surgery [7].

Finally, directly comparing postoperative short-term outcomes including the lengths of hospital and intensive care unit stay, duration of mechanical ventilation and 90-day mortality between patients with and without postoperative AKI or among patients with different postoperative AKI stage, was questionable, as their preoperative and intraoperative data that might affect postoperative short-term outcomes were significantly different. To obtain the true effects of AKI occurrence and severity on postoperative short-term outcomes in a retrospective study, the multivariate analysis or propensity score matching for adjustment of confounders is needed.

Thus, we argue that clarification of above issues will improve the transparency of this study design and be very helpful for interpretation of the findings.

## Data Availability

Not applicable.

## Abbreviations

SCr: Serum creatinine
AKI: Acute kidney injury
CPB: Cardiopulmonary bypass
